# A Low-Complexity DOA and Polarization Method of Polarization-Sensitive Array

**DOI:** 10.3390/s17051170

**Published:** 2017-05-20

**Authors:** Wen Dong, Ming Diao, Lipeng Gao, Lutao Liu

**Affiliations:** College of Information and Communication Engineering, Harbin Engineering University, Harbin 150001, China; a343720065@hrbeu.edu.cn (W.D.); diaoming@hrbeu.edu.cn (M.D.); liulutao@hrbeu.edu.cn (L.L.)

**Keywords:** DOA estimation, polarization estimation, polarization sensitive array (PSA), quaternion

## Abstract

This paper proposes a low-complexity method to estimate the direction of arrival and polarization based on the polarization sensitive array (PSA) which is composed of cross-dipoles. We built a half-quaternions model through the Cayley–Dickson form to remove the redundant information. Then, the directions of arrival (DOAs) were estimated via the root-MUSIC algorithm. Finally, the polarizations were estimated by generalized eigenvalue method. Unlike some existing searching algorithms, such as multiple signal classification (MUSIC), this method can avoid the peak searching and maintains high estimation accuracy. Moreover, we use the oblique projection operators to filter out the interference signals which are decoys of the target signal. Simulation results demonstrate the effectiveness and favorable performance of the proposed method.

## 1. Introduction

Polarization-sensitive arrays have received considerable attention in many disciplines, including multiple-input and multiple-output (MIMO) radar, sonar, and mobile communications [1,2,3,4,5,6]. Various array configurations have been proposed, including uniform linear array (ULA) [7] and uniform circular array (UCA) [8]. Moreover, the co-prime array [9,10,11] is also a significant configuration which consists of two uniform sparse linear subarrays. The direction of arrival (DOA) plays an important role in the processing of signals from these vector sensor arrays, which can make full use of the polarization diversity of the impinging sources during the last decades [12,13,14,15]. The most representative joint DOA and polarization estimation methods are based on the subspace algorithms, including the polarized multiple signal classification (MUSIC) method [16] and polarized signal parameters via rotational invariance technique (ESPRIT) method [17,18]. However, the polarized MUSIC method needs the peak searching which makes the computation complexity higher. Especially in polarization sensitive arrays (PSAs), the estimation of the 4-D parameter is a complex process. To solve this problem, an improved method called root-MUSIC has been proposed [19].

To reduce the computation complexity, several quaternion models have been studied in [20], including quaternions, half-quaternion, and biquaternions. In these models, quaternion is a supercomplex number which makes a multidimensional vector into a one-dimensional vector to reduce the computational effort. Miron et al. proposed a quaternion-MUSIC method [21] by using peak searching. A single polarized vector sensor is used in [22] to estimate the DOA and the polarization based on a new ESPRIT algorithm. The maximum likelihood (ML) algorithm [23] and [24] is an optimal algorithm for the DOA estimation that is much more complex than the other algorithms. Besides, various estimated DOA of coherent signal methods have been discussed, including the polarization difference smoothing (PDS) method [25] and polarization angular smoothing (PAS) [26]. In the electronic detection system, the detection of the presence of the decoy is the foundation of countering the radar active decoy. To detect the interference signal, the polarization estimation is an important processes. The decoys also have the same polarization information, which are different from target signal. In [27] , an oblique projection operator has been proposed which can filter out the decoy signals and retain the target signal.

However, it is shown in [16,21] that the MUSIC method can achieve a high accuracy by using peak searching. Hence, we need a method which can achieve a high accuracy and less complexity. To this end, we propose a joint DOA and polarization method for PSA. In the first step, we built a quaternion model to estimate the DOA. In the second step, the polarization information is estimated by the generalized eigenvector. Finally, we filter out the decoy signals via the oblique projection operator. Monte Carlo simulations verify the efficacy of the proposed method.

The remainder of this paper is organized as follows. The mathematical model and array configuration are given in Section 2. Section 3 presents the proposed DOA and polarization method. The computational complexity is discussed in Section 4. The performances of the proposed method in simulations are described in Section 5.

## 2. Problem Formulation

### 2.1. Quaternions

Quaternions are a four-dimensional hypercomplex number system, and they are widely used for the estimation of DOA and polarization parameters [21]. As an extension of complex numbers to four-dimensional (4-D) space, a quaternion *q* can be expressed as

(1) q=a+b·i+c·j+d·k

where a,b,c,d∈R,

(2) $$\begin{array}{c} i^{2}=j^{2}=k^{2}=ijk=-1\\ ij=-ji=k\\ ki=-ik=j\\ jk=-kj=i \end{array}$$

Several properties of quaternions are as follows: The conjugate of quaternion *q*, denoted $q^{*}$, is given

(3) $$q^{*}=a-b\cdot i-c\cdot j-d\cdot k$$

The norm of a quaternion is given by

(4) $$\left|q\right|=\sqrt{qq^{*}}=\sqrt{q^{*}q}=\sqrt{a^{2}+b^{2}+c^{2}+d^{2}}$$

and its inverse is:

(5) $$q^{-1}=\frac{q^{*}}{{\left|q\right|}^{2}}$$

By definition, some necessary equations can be obtained:

(6) $$h\cdot j=j\cdot h^{*}$$

(7) $$q^{*}q=qq^{*}$$

(8) $$q_{1}q_{2}\neq q_{2}q_{1}$$

(9) $${\left(q_{1}q_{2}\right)}^{*}=q_{2}^{*}q_{1}^{*}$$

where $h\in C,q_{1},q_{2}\in Q$ (Q is the quaternion field). The Cayley–Dickson form can be expressed as:

(10) q=α+β·j

where α=a+b·i,β=c+d·i. Through the Cayley-Dickson form, we can find that the quaternion is a supercomplex number with its real part and imaginary part all being complex numbers. This notation will be used in the following section to build the polarized signal model.

### 2.2. Array Configuration and Mathematical Model

**Figure 1:**
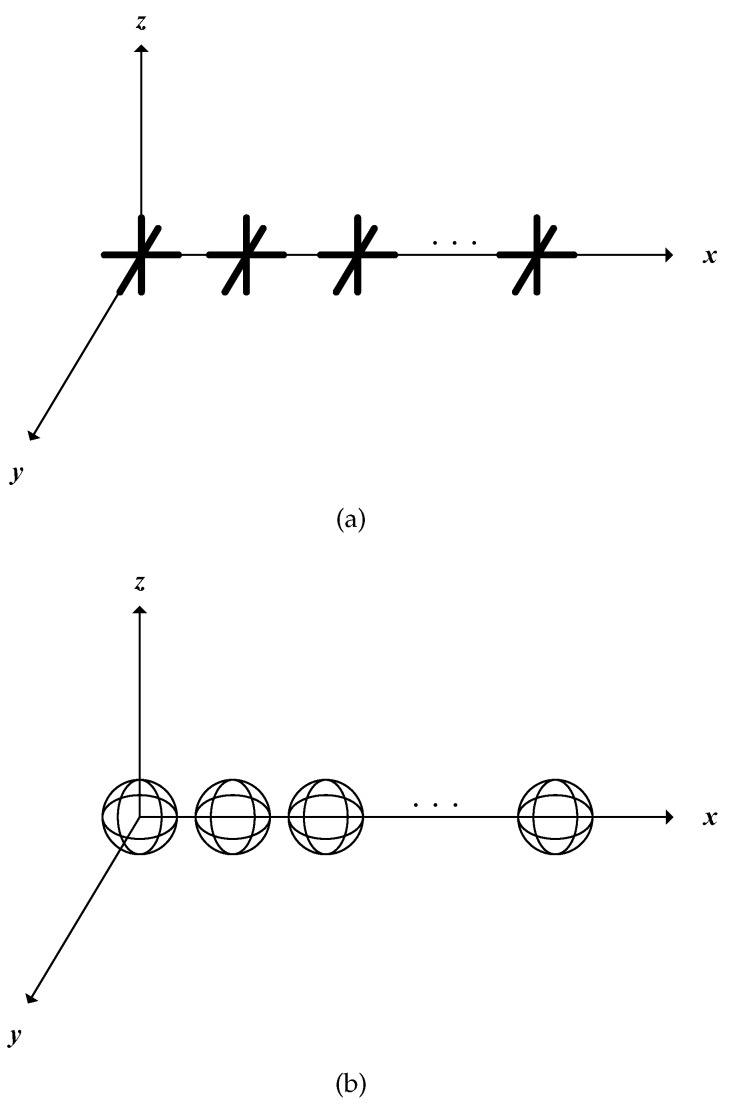
(**a**) Three orthogonally-oriented dipoles; (**b**) Three orthogonally-oriented dipoles.

PSA can acquire the polarization information besides the spatial information of the electromagnetic signals, which consists of a group of polarization-sensitive elements. Then, consider a vector sensor containing three electric and three magnetic orthogonal dipoles as discussed in [13].

(11) $$p=\left[\begin{array}{c} e_{x}\\ e_{y}\\ e_{z}\\ h_{x}\\ h_{y}\\ h_{z} \end{array}\right]=\left[\begin{array}{cc} -\sin\varphi & \cos\theta \cos\varphi \\ \cos\varphi & \cos\theta \sin\varphi \\ 0 & -\sin\theta \\ \cos\theta \cos\varphi & \sin\varphi \\ \cos\theta \sin\varphi & -\cos\varphi \\ -\sin\theta & 0 \end{array}\right]\left[\begin{array}{c} \cos\gamma \\ \sin\gamma e^{j\eta } \end{array}\right]$$

where 0≤θ<π/2 denotes the signal’s elevation angle, 0≤φ<2π denotes the azimuth angle, 0≤γ<π/2 represents the auxiliary polarization angle, and 0≤η<2π is the polarization phase difference. As demonstrated in [28], to estimate the DOA and polarization parameters, we need to obtain at least two of the six-component vector. In general, the vector sensor is composed of three orthogonally-oriented dipoles or loops, as depicted in Figure 1.

**Figure 2:**
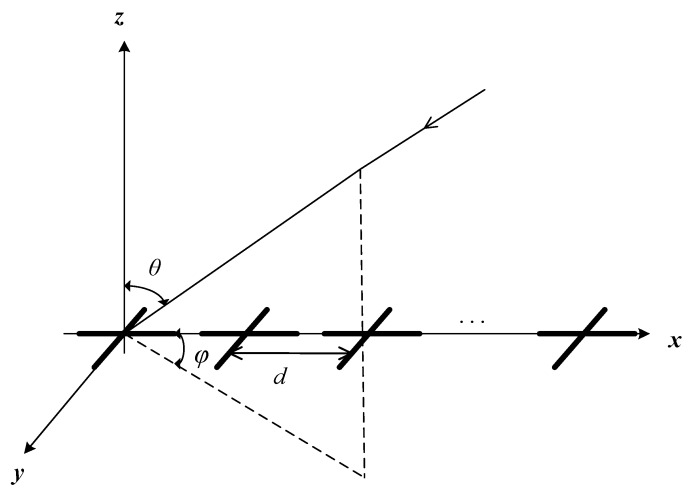
Uniform linear array configuration.

Note that the following analysis is adapted to all the polarized vector-sensor which can receive at least a two-dimensional electromagnetic vector. For convenience, we denote the orthogonal dipole parallel to the *x*-axis and *y*-axis, respectively, as depicted in Figure 2.

Consider *K* completely polarized narrow-band transverse electromagnetic (TEM) waves impinging on this array with *M* sensors. Then, each sensor can receive two electric field components ($e_{x}$ and $e_{y}$); thus, we can get the output of the *m*th array element at time *t* as follows:

(12) $$x_{m}\left(t\right)=\left[\begin{array}{c} x_{1m}\left(t\right)\\ x_{2m}\left(t\right) \end{array}\right]=\left[\begin{array}{c} \sum_{k=1}^{K}e_{x,k}\cdot e^{j\tau_{mk}}s_{k}\left(t\right)+n_{m}\left(t\right)\\ \sum_{k=1}^{K}e_{y,k}\cdot e^{j\tau_{mk}}s_{k}\left(t\right)+n_{m}\left(t\right) \end{array}\right]$$

where $x_{1m}$ and $x_{2m}$ are the output of antennas parallel to the *x*-axis and *y*-axis, respectively. $e_{x,k}$ and $e_{y,k}$ are given by Equation (11) with respect to the *k*th source. $\tau_{mk}$ is the phase shift associated with the *k*th signal’s propagation time delay between the *m*th element and the phase reference point, which is given by

(13) $$\tau_{mk}=\frac{2\pi d}{\lambda }\left(m-1\right)\sin\theta_{k}\cos\varphi_{k}$$

where $\theta_{k}$ is the elevation angles and $\varphi_{k}$ is the azimuth angles. *d* is the inter-sensor spacing, as depicted in Figure 1. Then, Equation (12) can be further rewritten as

(14) $$x\left(t\right)=\left[\begin{array}{c} x_{1}\left(t\right)\\ x_{2}\left(t\right) \end{array}\right]=\left[\begin{array}{c} AP_{1}s\left(t\right)+n_{1}\left(t\right)\\ AP_{2}s\left(t\right)+n_{2}\left(t\right) \end{array}\right]$$

where $s\left(t\right)={\left[s_{1}\left(t\right),s_{2}\left(t\right),\cdots ,s_{K}\left(t\right)\right]}^{T}$ and n(t) are the source and noise vectors, respectively. A is the M×K steering vector, and $P_{1}$, $P_{2}$ are given by

(15) $$\begin{array}{c} P_{1}=\left[\begin{array}{ccc} e_{x,1} & & \\ & \ddots & \\ & & e_{x,K} \end{array}\right]\\ P_{2}=\left[\begin{array}{ccc} e_{y,1} & & \\ & \ddots & \\ & & e_{y,K} \end{array}\right] \end{array}$$

The basic assumptions utilized throughout this paper are listed as follows.

(1) The K incoherent arriving signals s(t) are narrow band and circular signals, which means $E\{ss^{T}\}=\;0$.
(2) The entries of n(t) are white Gaussian noise and uncorrelated with each other. Noise from different sensors are independent, which means $E\left\{n_{1}n_{2}^{T}\right\}=E\left\{sn^{T}\right\}=E\left\{ns^{T}\right\}=E\left\{nn^{T}\right\}=0$.

## 3. Proposed Algorithm

In this section, a DOA and polarization estimation method is proposed based on the quaternion theory by using a uniform linear array.

### 3.1. Half-Quaternions Model

Through the Cayley-Dickson form, the half-quaternions model can be writen as

(16) $$x=x_{1}+x_{2}\cdot j$$

Then we can get the covariance matrix of *X* as follow

(17) $$\begin{array}{c} R=E\left\{xx^{H}\right\}=E\left\{\left(x_{1}+x_{2}\cdot j\right)\left(x_{1}^{H}-j\cdot x_{2}^{H}\right)\right\}\\ =E\{x_{1}x_{1}^{H}-x_{1}\cdot j\cdot x_{2}^{H}+x_{2}\cdot j\cdot x_{1}^{H}-x_{2}\cdot j\cdot j\cdot x_{2}^{H}\}\\ =E\{\Omega +\Psi \cdot j\} \end{array}$$

where $\Omega =x_{1}x_{1}^{H}+x_{2}x_{2}^{H}$, $\Psi =x_{2}x_{1}^{T}-x_{1}x_{2}^{T}$. ${\left(\cdot \right)}^{H}$ is the conjugate transpose matrix, and the quaternion mathematical operations can be found in Equations (6) to (9).

Through Equation (14), we can get

(18) $$\begin{array}{c} E\{\Psi \}\cdot j\\ =E\{\left(AP_{2}s+n_{2}\right){\left(AP_{1}s+n_{1}\right)}^{T}-\left(AP_{1}s+n_{1}\right){\left(AP_{2}s+n_{2}\right)}^{T}\}\cdot j\\ =E\{AP_{2}E\left\{ss^{T}\right\}{P_{1}}^{T}A^{T}+AP_{2}E\left\{sn_{1}^{T}\right\}+E\left\{n_{2}s^{T}\right\}{P_{1}}^{T}A^{T}+E\left\{n_{1}n_{2}^{T}\right\}\\ -AP_{1}E\left\{ss^{T}\right\}{P_{2}}^{T}A^{T}-AP_{1}E\left\{sn_{2}^{T}\right\}-E\left\{n_{1}s^{T}\right\}{P_{2}}^{T}A^{T}-E\left\{n_{1}n_{2}^{T}\right\}\}\cdot j \end{array}$$

where ${\left(\cdot \right)}^{T}$ denotes the transposed matrix. Due to the assumptions of signal as discussed in the Section 2.2, E{Ψ}·j=0.

Hence Equation (17) becomes

(19) $$\begin{array}{c} \begin{array}{c} R=E\{\Omega \}\\ =E\{\left(AP_{1}s+n_{1}\right){\left(AP_{1}s+n_{1}\right)}^{H}+\left(AP_{2}s+n_{2}\right){\left(AP_{2}s+n_{2}\right)}^{H}\}\\ =AP_{1}E\left\{ss^{H}\right\}P_{1}^{H}A^{H}+AP_{1}E\left\{sn_{1}^{H}\right\}+E\left\{n_{1}s^{H}\right\}P_{1}^{H}A^{H}+E\left\{n_{1}n_{1}^{H}\right\}\\ +AP_{2}E\left\{ss^{H}\right\}P_{2}^{H}A^{H}+AP_{2}E\left\{sn_{2}^{H}\right\}+E\left\{n_{2}s^{H}\right\}P_{2}^{H}A^{H}+E\left\{n_{2}n_{2}^{H}\right\}\\ =AP_{1}E\left\{ss^{H}\right\}P_{1}^{H}A^{H}+AP_{2}E\left\{ss^{H}\right\}P_{2}^{H}A^{H}+\sigma_{1}^{2}I+\sigma_{2}^{2}I \end{array} \end{array}$$

where $\sigma_{1}^{2}$ and $\sigma_{2}^{2}$ denote the noise variance. Then we define $E\left\{ss^{H}\right\}=diag\left\{\rho_{1}^{2},\rho_{2}^{2},\cdots ,\rho_{K}^{2}\right\}$ with $\rho_{k}^{2}$ is the power of the *k*th signal. Thus, we have

(20) $$\begin{array}{c} P_{1}E\left\{ss^{H}\right\}P_{1}^{H}=\left[\begin{array}{cccc} {∥e_{x,1}∥}^{2}\rho_{1}^{2} & & & \\ & {∥e_{x,2}∥}^{2}\rho_{2}^{2} & & \\ & & \ddots & \\ & & & {∥e_{x,K}∥}^{2}\rho_{K}^{2} \end{array}\right]\\ P_{2}E\left\{ss^{H}\right\}P_{2}^{H}=\left[\begin{array}{cccc} {∥e_{y,1}∥}^{2}\rho_{1}^{2} & & & \\ & {∥e_{y,2}∥}^{2}\rho_{2}^{2} & & \\ & & \ddots & \\ & & & {∥e_{y,K}∥}^{2}\rho_{K}^{2} \end{array}\right] \end{array}$$

where $∥\cdot ∥$ denotes the norm. Hence the covariance matrix R is written as

(21) $$R=A\left[\begin{array}{ccc} \left({∥e_{x,1}∥}^{2}+{∥e_{y,1}∥}^{2}\right)\rho_{1}^{2} & & \\ & \ddots & \\ & & \left({∥e_{x,K}∥}^{2}+{∥e_{y,K}∥}^{2}\right)\rho_{K}^{2} \end{array}\right]A^{H}+\left(\sigma_{1}^{2}+\sigma_{2}^{2}\right)I$$

Note that the noise is a linear superposition of two polarization dimensions, and the signal power is equivalent to the weightings. Due to E{Ψ}≠0 when the number of snapshot is limited, the information contained in E{Ψ} is redundant. Through Equation (19), the redundant information has been removed, hence this method can improve utilization of measurement data.

### 3.2. DOA Estimation

Without loss of generality, we assume that the azimuth angle of signals $\varphi =0^{\circ }$. Through Equation (19), the eigenvalue decomposition of covariance matrix can be obtained

(22) $$R=U_{S}\Lambda_{S}U_{S}^{H}+U_{N}\Lambda_{N}U_{N}^{H}$$

where $U_{S}$ and $U_{N}$ denotes the signal subspace and noise subspace, respectively. The diagonal matrix $\Lambda_{S}$ and $\Lambda_{n}$ are composed of *K* larger eigenvalues and M−K smaller eigenvalues, respectively. Invoking the MUSIC algorithm, we know

(23) $$\hat{\theta }=\arg\min_{\theta }a^{H}\left(\theta \right)U_{N}U_{N}^{H}a\left(\theta \right)$$

where a(θ) is the steering vector

(24) $$a\left(\theta \right)={\left[1,e^{j2\pi d\sin\theta /\lambda },\cdots ,e^{j2\pi Md\sin\theta /\lambda }\right]}^{T}$$

Substituting Equation (24) in Equation (23), we have

(25) $$\begin{array}{c} a^{H}\left(\theta \right)U_{N}U_{N}^{H}a\left(\theta \right)\\ =\sum_{m=0}^{M-1}\sum_{n=0}^{M-1}e^{2\pi md\sin\theta /\lambda }R_{N}\left(m,n\right)e^{-j2\pi nd\sin\theta /\lambda }\\ =\sum_{l=-(M-1)}^{M-1}e^{j2\pi ld\sin\theta /\lambda }\cdot r_{l} \end{array}$$

where $R_{N}=U_{N}U_{N}^{H}$ is a M×M matrix, and $r_{l}$ is given by

(26) $$r_{l}=\sum_{m-n=l}R_{N}\left(m,n\right)$$

Let $z=e^{j2\pi d\sin\theta /\lambda }$, then Equation (25) can be written as

(27) $$D\left(z\right)=\sum_{m-n=l}r_{l}z^{l}$$

Thus the estimation of Equation (23) has changed into the solution of D(z)=0. As $z=e^{j2\pi d\sin\theta /\lambda }$, the roots are supposed to lie on the unit circle. Then, the estimation of elevation angle is given by

(28) $$\hat{\theta }=\arcsin\left(\frac{\lambda }{2\pi d}\arg\left(z\right)\right)$$

### 3.3. Polarization Parameter Estimation

As can be seen from Equation (21), the polarization parameter become a weighting of the signal power, and hence we need reconstructing the covariance matrix as follow

(29) $$\begin{array}{c} \tilde{X}=\left[X_{1};X_{2}\right]\\ \tilde{R}=E\left\{\tilde{X}{\tilde{X}}^{H}\right\}\\ ={\tilde{U}}_{S}{\tilde{\Lambda }}_{S}{\tilde{U}}_{S}^{H}+{\tilde{U}}_{N}{\tilde{\Lambda }}_{N}{\tilde{U}}_{N}^{H} \end{array}$$

Equation (23) can be written as follow

(30) $$\begin{array}{c} \left\{\hat{\gamma },\hat{\eta }\right\}=\arg\min_{\gamma ,\eta }\left\{{\left(\frac{D_{\hat{\theta }}h_{\gamma ,\eta }}{∥D_{\hat{\theta }}h_{\gamma ,\eta }∥}\right)}^{H}{\tilde{U}}_{N}{\tilde{U}}_{N}^{H}\left(\frac{D_{\hat{\theta }}h_{\gamma ,\eta }}{∥D_{\hat{\theta }}h_{\gamma ,\eta }∥}\right)\right\}\\ \;\;=\arg\min_{\gamma ,\eta }\left\{\frac{h_{\gamma ,\eta }^{H}H\left(\hat{\theta }\right)h_{\gamma ,\eta }}{h_{\gamma ,\eta }^{H}D_{\hat{\theta }}^{H}D_{\hat{\theta }}h_{\gamma ,\eta }}\right\}\\ \;\;=\arg\min_{h_{\gamma ,\eta }\neq 0}\left\{\frac{h_{\gamma ,\eta }^{H}H\left(\hat{\theta }\right)h_{\gamma ,\eta }}{h_{\gamma ,\eta }^{H}D_{\hat{\theta }}^{H}D_{\hat{\theta }}h_{\gamma ,\eta }}\right\}\\ \;\;=\arg\min_{h_{\gamma ,\eta }^{H}D_{\hat{\theta }}^{H}D_{\hat{\theta }}h_{\gamma ,\eta }=1}\left\{\frac{h_{\gamma ,\eta }^{H}H\left(\hat{\theta }\right)h_{\gamma ,\eta }}{h_{\gamma ,\eta }^{H}D_{\hat{\theta }}^{H}D_{\hat{\theta }}h_{\gamma ,\eta }}\right\} \end{array}$$

where $D_{\hat{\theta }}$ is the 2M×2 matrix only with respect to θ.

(31) $$\begin{array}{c} D_{\hat{\theta }}=a\left(\hat{\theta }\right)⊗\Xi \left(\hat{\theta }\right),H\left(\hat{\theta }\right)=D_{\hat{\theta }}^{H}{\tilde{U}}_{N}{\tilde{U}}_{N}^{H}D_{\hat{\theta }}\\ a\left(\hat{\theta }\right)={\left[1,e^{j2\pi d\sin\hat{\theta }/\lambda },\cdots ,e^{j2\pi Md\sin\hat{\theta }/\lambda }\right]}^{T}\\ \Xi \left(\hat{\theta }\right)=\left[\begin{array}{cc} -1 & 0\\ 0 & \cos\hat{\theta } \end{array}\right]\\ h_{\gamma ,\eta }=\left[\begin{array}{c} \cos\gamma \\ \sin\gamma e^{j\eta } \end{array}\right] \end{array}$$

where $a(\hat{\theta })$ is the M×1 steering vector of the ULA. $\Xi (\hat{\theta })$ and $h_{\gamma ,\eta }$ can be obtained from Equation (11). Note that $\varphi =0^{\circ }$ in the ULA and $\hat{\theta }$ has been estimated in Section 2.2.

Equation (30) can be further rewritten as

(32) $$\begin{array}{c} \min_{h_{\gamma ,\eta }}h_{\gamma ,\eta }^{H}H\left(\theta \right)h_{\gamma ,\eta }\\ s.t.\;h_{\gamma ,\eta }^{H}D_{\theta }^{H}D_{\theta }h_{\gamma ,\eta }=1 \end{array}$$

This optimization problem can be solved by Lagrange multiplier method as follow

(33) $$\Upsilon_{\theta }\left(h_{\gamma ,\eta },\mu \right)={h_{\gamma ,\eta }}^{H}H\left(\theta \right)h_{\gamma ,\eta }+\mu \left(1-{h_{\gamma ,\eta }}^{H}D_{\theta }^{H}D_{\theta }h_{\gamma ,\eta }\right)$$

Taking a derivative by $h^{*}$ and μ, then we can get

(34) $$\begin{array}{c} \left\{\begin{array}{c} H\left(\theta \right)h_{\gamma ,\eta }=\mu D_{\theta }^{H}D_{\theta }h_{\gamma ,\eta }\\ h_{\gamma ,\eta }^{H}D_{\theta }^{H}D_{\theta }h_{\gamma ,\eta }=1 \end{array}\right.\\ \Rightarrow h_{\gamma ,\eta }^{H}H\left(\theta \right)h_{\gamma ,\eta }=\mu =\frac{h_{\gamma ,\eta }^{H}H\left(\theta \right)h_{\gamma ,\eta }}{h_{\gamma ,\eta }^{H}D_{\theta }^{H}D_{\theta }h_{\gamma ,\eta }}\geq 0 \end{array}$$

From Equation (34), we can know that $\min(h_{\gamma ,\eta }^{H}H\left(\theta \right)h_{\gamma ,\eta })=\min\left(\mu \right)$. μ is the generalized eigenvalue of matrix pencil $\left\{H\left(\theta \right),D_{\theta }^{H}D_{\theta }\right\}$, and $h_{\gamma ,\eta }$ is the generalized eigenvector. Hence the eigenvectors corresponding to the smallest eigenvalue is the estimation of polarization parameters.

(35) $$\begin{array}{c} {\hat{h}}_{\gamma ,\eta }=\hbar_{\min}\left\{H\left(\hat{\theta }\right),D_{\hat{\theta }}^{H}D_{\hat{\theta }}\right\}\\ \hat{\gamma }=\arctan\left\{\left|{\hat{h}}_{\gamma ,\eta }\left(2\right)/{\hat{h}}_{\gamma ,\eta }\left(1\right)\right|\right\}\\ \hat{\eta }=\arg\left\{{\hat{h}}_{\gamma ,\eta }\left(2\right)/{\hat{h}}_{\gamma ,\eta }\left(1\right)\right\} \end{array}$$

where $\hbar_{\min}\left\{\cdot \right\}$ denotes the eigenvectors corresponding to the smallest eigenvalue.

### 3.4. Oblique Projection Operators

In a practical application, we need to enhance signals while nulling interferences. Hence the oblique projection operator was proposed in [27]. We assume that all the doa and polarization parameters of signals have been estimated.

(36) $$a_{s}=\left[\begin{array}{c} 1\\ e^{j2\pi d\sin\theta_{0}/\lambda }\\ \vdots \\ e^{j2\pi Md\sin\theta_{0}/\lambda } \end{array}\right];w_{i}=\left[\begin{array}{c} 1\\ e^{j2\pi d\sin\theta_{i}/\lambda }\\ \vdots \\ e^{j2\pi Md\sin\theta_{i}/\lambda } \end{array}\right],\;i=1,\cdots ,K^{\prime }$$

where $a_{s}$ is the steering vector of the target signal, and $w_{i}$ is the the steering vector of the *i*th interference ($1+K^{\prime }=K$). Then the oblique projection operators are given by

(37) $$\begin{array}{c} P_{w_{i}}=I-w_{i}{\left(w_{i}^{H}w_{i}\right)}^{-1}w_{i}^{H}\\ E_{sw_{i}}=s{\left(a_{s}^{H}P_{w_{i}}a_{s}\right)}^{-1}a_{s}^{H}P_{w_{i}} \end{array}$$

where $P_{w}$ and $E_{sw}$ are the orthogonal projection operators and oblique projection operators, respectively. The array output is written as

(38) $$X^{\prime }\left(t\right)=\left[\begin{array}{c} E_{sw_{1}}\cdots E_{sw_{K^{\prime }}}X_{1}\left(t\right)\\ E_{sw_{1}}\cdots E_{sw_{K^{\prime }}}X_{2}\left(t\right) \end{array}\right]$$

Now the received data $X^{\prime }\left(t\right)$ that only contains the information of the target signal has been gotten. The main steps of the proposed method are summarized in Algorithm 1 as follow.

**Algorithm 1** Steps in the Proposed Method**Input**: X(1),X(2),⋯,X(N)1. obtain X according to Equation (11)**DOA Estimation**:2. Calculate the covariance matrix R=E{Ω} via Equation (8)3. Divide R into $U_{S}$ and $U_{N}$ according to Equation (19)4. Calculate $r_{l}$ according to Equation (23)5. Calculate the roots of $r_{l}$ which lie on the unit circle6. The estimates of DOA ($\hat{\theta }$) are obtained from Equation (25)**Polarization Parameter Estimation**:7. Calculate the covariance matrix $\tilde{X}=\left[X_{1};X_{2}\right]$ and the noise subspace ${\tilde{U}}_{N}$ via Equation (26)8. Calculate $D_{\hat{\theta }}$ and $H(\hat{\theta })$ according to Equation (28)9. Obtain the generalized eigenvectors corresponding to the smallest eigenvalue from Equation (32)10. Estimate Polarization Parameters ($\hat{\eta }$ and $\hat{\gamma }$) via Equation (32)
**Oblique Projecting Filter**
11. Find out the target signal $A_{S}$ and the interferences W through Equation (33)12. Compute the oblique projection operators via Equation (34)13. Filter out interfering signals using Equation (35)

## 4. Computational Complexity

The complexity of the algorithm mainly depends on two aspects: First, the the maximum likelihood estimation of the covariance matrix. Second, the process of peak searching. To demonstrate the advantage of the proposed method, we discuss the computational complexities of the proposed method and the traditional polarization MUSIC algorithm.

Table 1 presents the comparison of computational complexities of the two methods. *N* denotes the snapshot number, and $\Delta_{s}$ denotes the number of searching points.

As can be seen from Table 1, the proposed method based on quaternions has a lower computational complexity. That is because the covariance matrix $R\left(X\right)\in C^{2N\times 2N}$ has become $R\left(\Omega \right)\in C^{N\times N}$ via Equation (16). As discussed in Section 3.2 and Section 3.3, we use the root algorithm and the generalized eigenvector algorithm to estimate the DOA and polarization parameters without peak searching. Furthermore, the advantage of the proposed method becomes increasingly obvious on the array which can estimate 2-D DOA information.

## 5. Simulation

In this section, we compare the proposed method with the standard root-MUSIC [13] and the Cramér-Rao lower bound (CRB) is also used as a benchmark [29]. Consider a ULA which contains a total of M=8×2 dipoles and the inter-sensor spacing d=0.5λ. Two-hundred independent Monte Carlo trials are conducted for the following simulations, and the root mean squared error (RMSE) is chosen as a performance metric with different SNR, which are defined as

(39) $$RMSE=\sqrt{\frac{1}{200K}\sum_{i=1}^{200}\sum_{k=1}^{K}({\hat{\theta }}_{k}-\theta_{k}{)}^{2}}$$

(40) $$SNR=10\log_{10}\frac{\rho^{2}}{\sigma^{2}}$$

**Figure 3:**
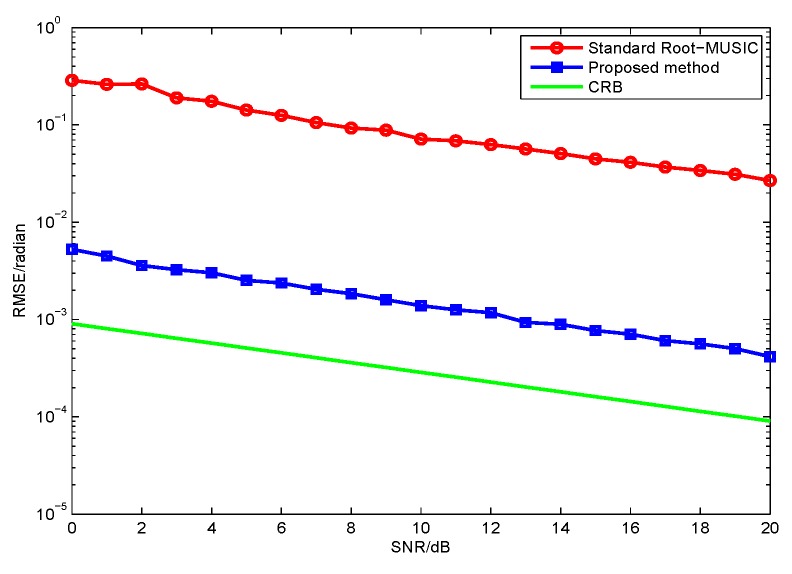
RMSE versus of θ estimates signal-to-noise ratio (SNR) for two methods with fixed snapshot number 1000.

**Figure 4:**
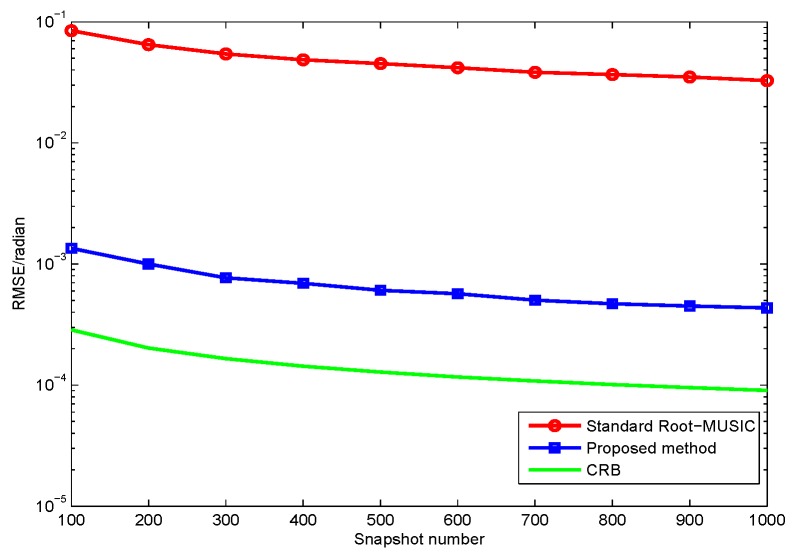
RMSE versus θ estimates snapshot number for two methods with fixed SNR of 20 dB.

In the first simulation, we evaluate the DOA estimation performance of the proposed method with different SNR and snapshot number. Assume that the four far-field narrowband completely polarized electromagnetic wave sources impinge on the array as depicted in Figure 2. We set $\left\{\theta_{1}=5^{\circ },\gamma_{1}=5^{\circ },\eta_{1}=11^{\circ }\right\},\left\{\theta_{2}=16^{\circ },\gamma_{2}=12^{\circ },\eta_{2}=34^{\circ }\right\},\left\{\theta_{3}=28^{\circ },\gamma_{3}=26^{\circ },\eta_{3}=74^{\circ }\right\}$, and $\left\{\theta_{4}=40^{\circ },\gamma_{4}=34^{\circ },\eta_{4}=124^{\circ }\right\}$. The snapshot number and the wavelength are set to be 1000 and λ=0.13, respectively. Figure 3 depicts the performance versus SNR with snapshot number = 1000, and Figure 4 depicts the performance versus snapshot number SNR = 20 dB. It can be seen from Figure 3 and Figure 4 that the proposed method has much better performance than the standard root-MUSIC.

**Figure 5:**
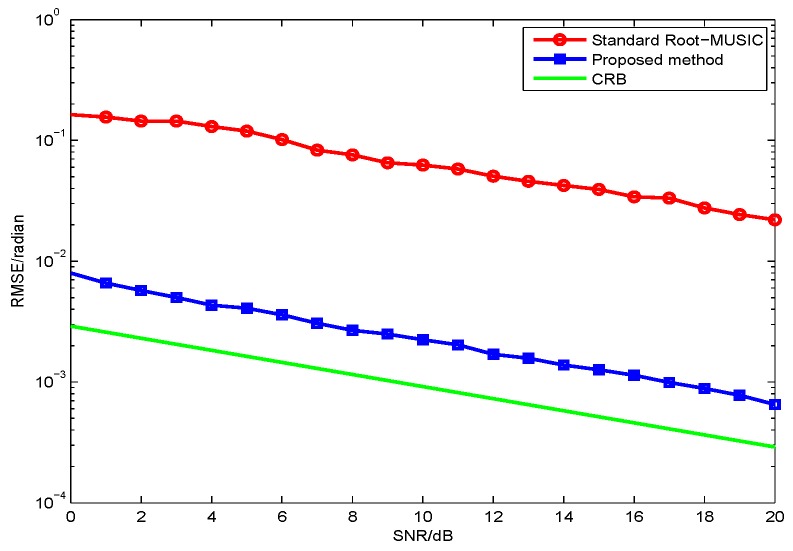
RMSE of γ estimates versus SNR with snapshot number 1000.

**Figure 6:**
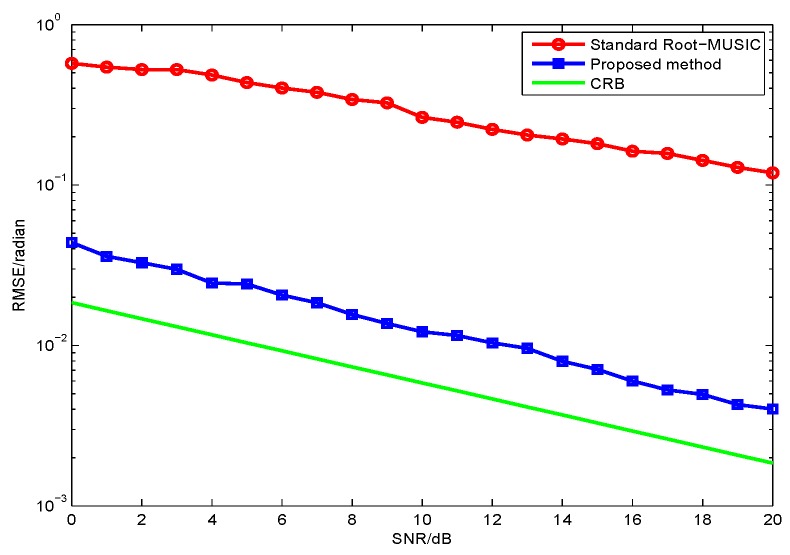
RMSE of η estimates versus SNR with snapshot number 1000.

In the second simulation, we evaluate the polarization estimation performance of the proposed method with different SNR. As discussed in the first simulation, the proposed method has a better DOA estimation performance. The proposed method have a lower RMSE of the polarization estimation, because the polarization estimation of the two methods are all based on their DOA estimation. Hence, we only need to compare the proposed method with CRB as in Figure 5 and Figure 6. The results from Figure 3, Figure 4, Figure 5 and Figure 6 demonstrate that the proposed method yields more accurate DOA and polarization estimates than the standard root-MUSIC.

**Figure 7:**
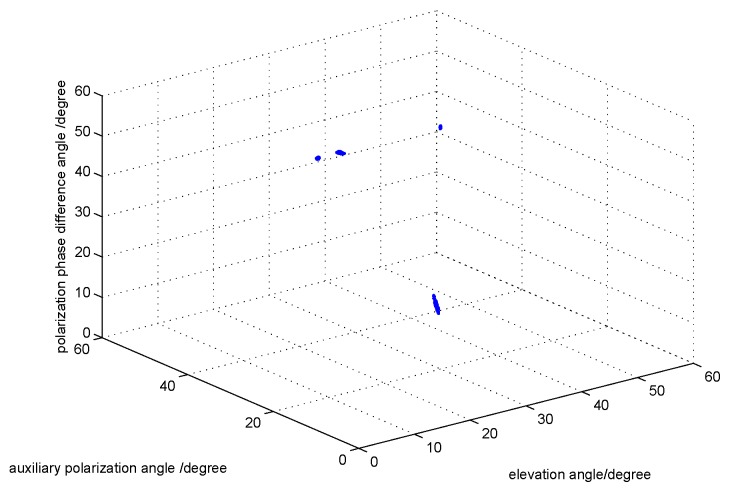
The DOA and polarization estimation before the filtering with fixed SNR 20.

**Figure 8:**
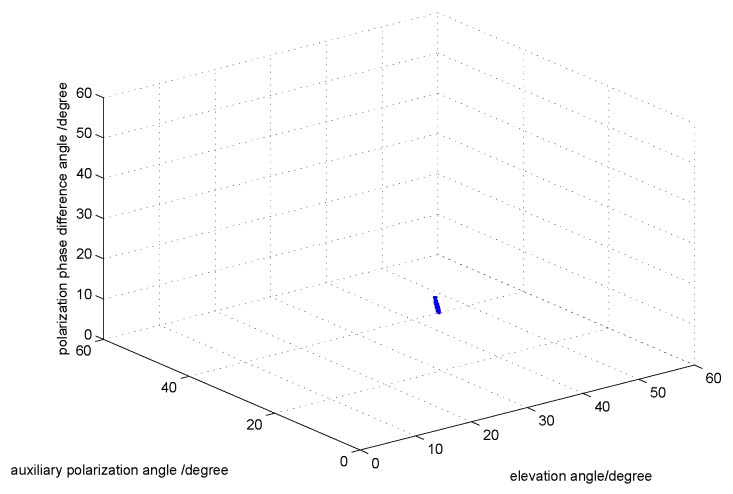
The DOA and polarization estimation after the filtering with fixed SNR 20.

The third simulation compares the estimation performance of the oblique Projection Operators. For comparison purposes, we set a target signal and three interference signals. The target signal is parameterized by $\left\{\theta_{1}=20^{\circ },\gamma_{1}=8^{\circ },\eta_{1}=25^{\circ }\right\}$, and the three interference signals are parameterized by $\left\{\theta_{2}=16^{\circ },\gamma_{2}=20^{\circ },\eta_{2}=60^{\circ }\right\},\left\{\theta_{3}=30^{\circ },\gamma_{1}=20^{\circ },\eta_{1}=60^{\circ }\right\}$, and $\left\{\theta_{4}=8^{\circ },\gamma_{4}=20^{\circ },\eta_{4}=60^{\circ }\right\}$. Note that the three interference signals have the same polarization parameters, which are different from the target signal. By comparing Figure 7 and Figure 8, we can find that the interference signals have been filtered out.

## 6. Conclusions

In this paper, we have proposed a low-complexity DOA and polarization estimation method based on quaternions for PSA. We firstly built a mode of quaternions to remove the redundant information, which can reduce the complexity. Then, we obtain the DOA and polarization parameters by the root-MUSIC and generalized eigenvalue methods. Finally, the interference signals are filtered out through the oblique projection operators, and then we can obtain the target signal only. Simulation results show that the proposed method has a better performance with a low-complexity.

## Figures and Tables

**Table 1:** Comparison of computational complexity of two methods.

Methods	Covariance Matrix	Peak Search
Proposed	$M^{2}N$	without
MUSIC	${\left(2M\right)}^{2}N$	$\left(4M\;+\;4\right)\;\times \;2M\;\times \;\Delta_{s}$
